# Galectin-7 Expression Potentiates HER-2-Positive Phenotype in Breast Cancer

**DOI:** 10.1371/journal.pone.0166731

**Published:** 2016-11-30

**Authors:** Andrée-Anne Grosset, Françoise Poirier, Louis Gaboury, Yves St-Pierre

**Affiliations:** 1 INRS-Institut Armand-Frappier, Laval, QC, Canada; 2 IRIC | Université de Montréal, Montreal, QC, Canada; 3 Institut Jacques Monod, CNRS, Univ Paris Diderot, Sorbonne Paris Cité, Paris, France; University of Alabama at Birmingham, UNITED STATES

## Abstract

HER-2 positive tumors are among the most aggressive subtypes of breast cancer and are frequently associated with metastasis and poor outcome. As with other aggressive subtypes of breast cancer, these tumors are associated with abnormally high expression of galectin-7 (gal-7), which confers metastatic breast tumor cells with increased invasive behavior. Although previous studies in the rat model of breast tumorigenesis have shown that gal-7 is also increased in primary breast tumor, its contribution to the development of the primary breast tumors remains unclear. In the present work, we have used genetically-engineered gal-7-deficient mice to examine the role of gal-7 in the development of the mammary gland and of breast cancer. Using histological and immunohistological analysis of whole mammary glands at different stages of development, we detected no significant changes between normal and gal-7-deficient mice. To test the involvement of gal-7 in breast cancer, we next examined the effects of loss of gal-7 on mammary tumor development by crossing gal-7-deficient mice with the mammary tumor transgenic mouse strain FVB-Tg(MMTV-Erbb2)NK1Mul/J. Finally, assessment of mice survival and tumor volume showed a delay of mammary tumor growth in the absence of systemic gal-7. These data suggest that gal-7 could potentiate the phenotype of HER-2 positive primary breast cancer.

## Introduction

Breast cancer is among the most commonly diagnosed cancer among women and is a major cause of cancer-associated mortality [1]. Historically classified according to histomorphological features, breast cancer is now considered a group of highly heterogeneous diseases that can be better distinguished at the molecular level according to hormone receptor status, HER-2 gene over-expression or amplification, the fraction of proliferative cells or on gene expression profile [2, 3]. Tumors with increased levels of HER-2 are referred to HER-2-positive breast cancer. They represent approximately 20% all breast cancer cases and harbor Erbb2 gene amplification, resulting in greater than normal amounts of the HER-2 protein [4]. Together with triple-negative breast cancer, they tend to grow and spread more aggressively than other breast cancers and are considered among the most aggressive subtypes of breast cancer. A considerable amount of knowledge on the biological and molecular features of these cancers has been obtained using a number of transgenic mouse models, including the development of mice harboring an Erbb2 gene under the control of the MMTV promoter. Overexpression of this gene has been shown to induce multifocal tumors at an average age of seven months [5]. Signaling pathways regulated by HER-2 have also been identified through the use of MMTV-Erbb2 mice. Not surprisingly, such mouse models are commonly used to study the role of other proteins in HER-2 positive breast carcinomas.

Galectins belongs to a family of widely expressed proteins that contains 15 members, named according to the order of their discovery [6, 7]. All galectins share an approximately 130 amino acid-long conserved sequence that encodes a carbohydrate recognition domain (CRD) with affinity to β-galactosides. Binding of galectins on cell surface N- or O-linked glycans is well-known to induce the formation of lattices that regulate surface retention and signaling threshold of cell surface glycoreceptors [8]. This has been well established for galectin-1 (gal-1) and gal-3. For example, lattice formation following binding of gal-3 to β-1,6-*N*-acetylglucosamine branched glycans leads to alterations in the clustering of membrane glycoreceptors, resulting in functional advantages for tumor cells [9]. Not surprisingly, alterations in the expression level of gal-3 plays a central role in modulating tumor progression, most notably in the case of breast cancer where gal-3 is expressed at abnormally high levels in both cancer and stromal cells [10–14].

Most of our knowledge on the role of galectin in breast cancer derives from studies that focused on gal-1 and gal-3, the most studied members of the galectin family. There is increasing evidence, however, that other members of the galectin family are also important in tumor progression. We have recently shown that multiple galectins are expressed in highly aggressive breast cancer tissues [15]. In the case of gal-7, we and others have reported that this gene is expressed at high levels in aggressive subtypes of breast cancer, including HER-2-positive subtype [2, 16, 17]. The expression of gal-7 in breast cancer cells confers increased metastatic behavior, suggesting that gal-7 plays a central role in late stages of the disease. Using a mouse model, we have shown that ectopic expression of gal-7 in breast cancer cells increases their ability to form bone and lung metastasis. In humans, we found that high levels of gal-7 expression correlates with lymph node metastasis in patients with HER-2-positive breast carcinoma [16]. Whether gal-7 modulates the early stage of the disease, however, remains unclear. Yet, there are indications that gal-7 could modulate the onset of the disease. For example, gal-7 is constitutively expressed in myoepithelial cells of the adult mammary gland [16, 18]. Lu *et al*. have also shown that gal-7 expression is rapidly induced in chemically-induced rat mammary tumors [19]. In the present work, we have investigated the role of gal-7 in regulating mammary tumor development using gal-7-deficient mice (KOG7) and the mouse model of breast cancer FVB-Tg(MMTV-Erbb2)NK1Mul/J in which an activated form of Erbb2 is specifically expressed in mammary tissue. Our results show that genetic ablation of gal-7 does not significantly impair the normal development of the mammary gland. In the absence of gal-7, however, mice with mammary tumors survive significantly longer and display primary mammary tumors of reduced sizes in comparison to mice expressing gal-7.

## Materials and Methods

### Mice

C57BL/6 mice and FVB-Tg(MMTV-ErbB2)NK1Mul/J transgenic mice [20] were obtained from The Jackson Laboratory (Bar Harbor, ME). *C57BL/6-lgals7*-deficient (KOG7) mice have been described [21]. Mice were maintained on a 12 hour light and dark cycle on a normal chow diet. The mice had access to water and diet *ad libitum*. The MMTV-ErbB2 mice were crossed with KOG7 mice. F1 mice were bred with *lgals7*^+/−^ female mice to obtain MMTV-ErbB2^+/−^/lgals7^pos^, and MMTV-ErbB2^+/−^/lgals7^neg^ littermates. All experiments were conducted using virgin females. and approved by the Institutional Animal Care and Use Committee (CISAU) of INRS-Institut Armand-Frappier (Protocols #0702–08 and #0706–01). Mice were monitored twice a week until tumors developed after which they were monitored daily. Body weight was measured weekly. Tumor development was monitored by measuring length and width with calipers. The following humane endpoints were used and approved by the institutional animal care committee: signs of lethargy, weakness, hunched posture, rough hair coat, decreased mobility, rapid or labored breathing, or if there was weight loss of more than 20% of body weight. Mice also euthanized if tumors reached 2 cm^3^. All animals were euthanized using CO_2_ narcosis prior to collection and fixation of tissues. Statistical analyses of tumor growth curves were conducted on the natural log of tumor volumes in the two groups and compared with controls using unpaired *t* test.

### Genotyping by PCR

DNA was extracted from the end of the tail. *ErbB2* transgene (sense primer: 5’- CCC CGG GAG TAT GTG AGT GA -3’ and antisense primer: 5’- ACA GTC GGA AGT TTT GTC GAG T -3’), *galectin-7* (sense primer: 5’- GGG CTT TGT GGG AAT ATT GAT AAC C -3’ and antisense primer: 5’- GGT ACA TTT GGA CGA TAC GCC ACT C -3’), *Neo-A3* (sense primer: 5’- TCA TTA TTT GAC CCT CCG TTA CTG G -3’ and antisense primer: 5’- GCA CTG TTT ACC TTC ATC GTG CAG A -3’) and *GAPDH* (sense primer: 5’- CGT CGT GGA TCT GAC GTG CCG -3’ and antisense primer: 5’- GGG GTC GTT CCT GTG ACT CGT T -3’) were amplified using the following conditions: 94°C for 3 min, followed by 39 cycles of the following: 94°C for 1 min, 60°C for 1 min, and 72°C for 1 min, followed by a final extension step at 72°C for 10 min. PCR was performed in a thermal cycler (Eppendorf, Mississauga, ON, Canada). The amplified products were analyzed by electrophoresis using 1% agarose gels and SYBR Safe (Life Technologies) staining and UV illumination.

### Cell Lines and Reagents

The MCF-7 neo (clone 1) and MCF-7 HER-2 (clone 18) cell lines were a generous gift from Dr. Mien-Chie Hung (The University of Texas MD Anderson Cancer Center, Houston, TX). These cells were maintained in Dulbecco’s Modified Eagle’s Medium (DMEM) high glucose / F12 (1:1) supplemented with 10% (v/v) FBS, 2 mM L-glutamine and 10 mM HEPES buffer. All cell culture products were purchased from Life Technologies (Burlington, ON, Canada). Immunohistochemical reactions on smear cells were realized as described below.

### RNA isolation and RT-PCR

Total cellular RNA was isolated from cells using the TRIzol reagent (Life Technologies) according to the manufacturer’s instructions. First-strand cDNA was prepared from 2 μg of cellular RNA in a total reaction volume of 20 μL using the reverse transcriptase Omniscript (QIAGEN, Mississauga, ON, Canada). After reverse transcription, human *galectin-7* (gene ID 3963, sense primer: 5’- ATG TCC AAC GTC CCC CAC AAG -3’ and antisense primer: 5’- CTC CAC GAG TAG TAG CGC AGT -3’) and *GAPDH* (gene ID 2597, sense primer: 5’- CGG AGT CAA CGG ATT TGG TCG TAT -3’ and antisense primer: 5’-CAG AAG TGG TGG TAC CTC TTC CGA -3’) cDNAs were amplified using the following conditions: 94°C for 3 min, followed by 35 cycles of the following: 94°C for 1 min, 62°C for 1 min, and 72°C for 1 min, followed by a final extension step at 72°C for 10 min. PCR was performed in a thermal cycler (Eppendorf, Mississauga, ON, Canada). The amplified products were analyzed by electrophoresis using 1% agarose gels and SYBR Safe (Life Technologies) staining and UV illumination.

### Histological Analysis

Whole mammary glands collected at different stages of development were formalin-fixed and paraffin-embedded. Then, sections of 4 μm were stained with hematoxylin and eosin and scanned at a high resolution using the Nanozoomer Digital Pathology (Hamamatsu, Bridgewater, NJ).

### Tissue Microarrays and Immunohistochemistry

Formalin-fixed paraffin-embedded material from each primary tumor sample and normal mammary gland was used to construct tissue microarrays with an automated arrayer designed to construct high-density tissue microarray blocks (ATA-27 Beecher Instruments, Sun Prairie, WI). To that end, triplicate 1 mm cores from each tumor and control tissues were punched out and arrayed into two recipient blocks. For immunohistochemical analysis, three-micrometer thick sections were prepared from each TMA. Immunostaining reactions were carried out using the Discovery XT automated immunostainer (Ventana Medical Systems, Tucson, AZ). Deparaffinated sections were incubated in cell conditioning buffer pH 8 for antigen retrieval and then with primary antibodies for 1 to 3 hrs: goat polyclonal anti-galectin-7 (1:100; R&D Systems, Minneapolis, MN), rabbit monoclonal anti-estrogen receptor α (1:50; EMD Millipore, Darmstadt, Germany), rabbit polyclonal anti-keratin 5 (1:100; Biolegend, San Diego, CA), rabbit polyclonal anti-HER-2 (1:100; Thermo Fisher Scientific, Waltham, MA), rabbit monoclonal anti-Ki-67 (1:1000; Biocare Medical, Concord, CA). The slides were counterstained with hematoxylin and bicarbonate. Each section was scanned at a high resolution using the Nanozoomer whole-slide cell scanner (Hamamatsu, Bridgewater, NJ).

### Statistical analysis

Fisher’s exact test, *t* test and Kaplan-Meier curves were assessed using GraphPad Prism 5.00 (GraphPad Software, San Diego, CA). A *p* value of 0.10 or less was considered statistically significant.

## Results and Discussion

### Mammary gland development in gal-7-deficient mice

**Fig 1:**
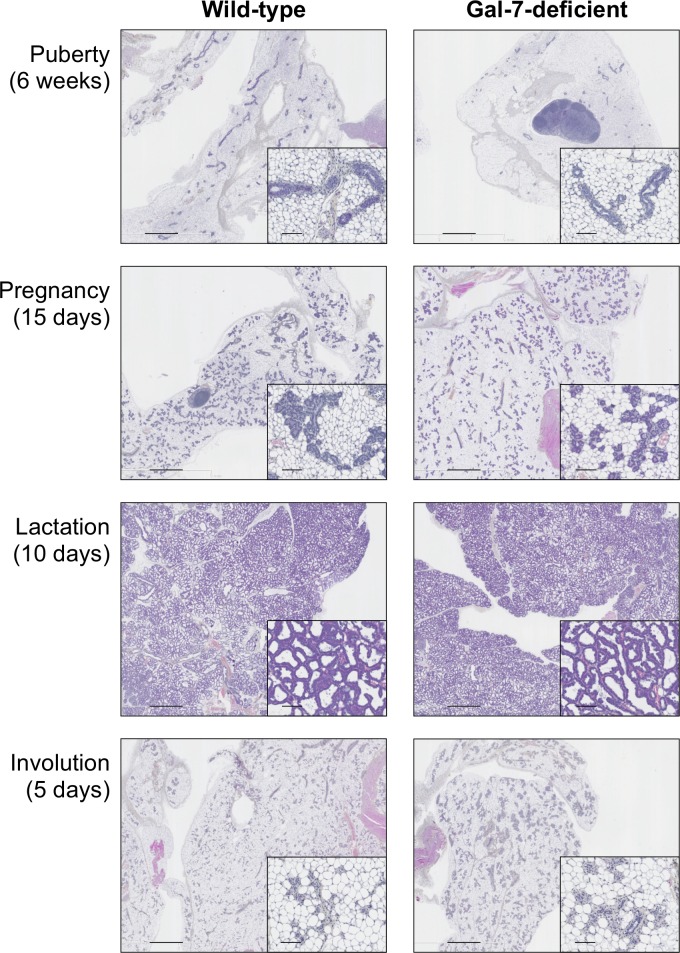
No significant effects on mammary gland developmental stages in the absence of gal-7. Histology assessment of whole mammary glands (H&E stained) of wild-type and galectin-7-deficient mice. Three mice were used for each group of mammary gland at three distinct stages: puberty (6 weeks of age), pregnancy (5, 10 and 15 days), lactation (0, 5 and 10 days) and involution (1 and 5 days). Scale bars, 1 mm and 100 μm (insets).

**Fig 2:**
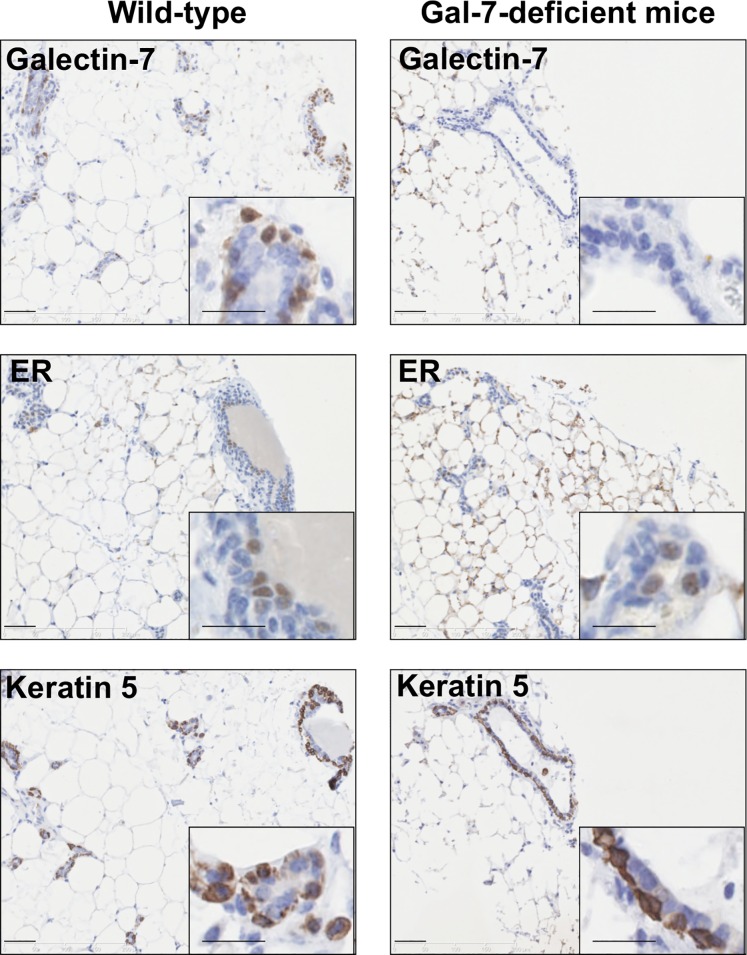
Expression of mammary epithelial markers in wild-type and galectin-7-deficient mice. Immunohistochemical detection of gal-7, estrogen receptor (ER; mammary luminal epithelial cell marker) and cytokeratin 5 (CK5; mammary myoepithelial cell marker) on normal mammary tissues of wild-type and galectin-7-deficient mice. Scale bars, 50 μm and 25 μm (insets).

Previous studies have shown that gal-7-deficient mice live and reproduce normally, at least in animal house conditions [21]. Observations over several years have also failed to reveal any obvious abnormalities with regards to feeding of pups, litter size or body weights of pups during lactation. However, because on one hand gal-7 is constitutively expressed in mammary myoepithelial cells and is an important regulator of cell homeostasis [16, 21–23], and, on the other, given the key role of myoepithelial cells during mammary gland development [24], we carried out a histological analysis of the mammary tissues at different stages of development to determine whether absence of gal-7 may alter the epithelial integrity of the mammary gland. Our results showed no detectable difference in pubertal mammary gland between virgin *lgals7*^−/−^ and age-matched wild-type (WT) female mice. No differences could be detected in mice at 15 days of pregnancy, during lactation or post-lactational involution stage (**Fig 1**). Furthermore, the absence of gal-7 did not prevent the epithelial expression of the estrogen receptor (13 and 17% of positive cells in control and gal-7deficient mice respectively; *p* = 0.67) (Fig 2). Similarly, cytokeratin 5 (CK5), a marker of mammary myoepithelial cells, was expressed in virtually all epithelial cells in both wild-type and gal-7-deficient mice. Our results are somewhat similar to those of Gendronneau *et al*. who showed that absence of gal-7 does not affect the expression profiles of markers of different epidermis layers, such as CK5, CK10 and loricrin [21]. They also support the view that deletion of a single galectin has limited impact in the overall development of mice, at least for the cases of gal-1, gal-3, gal-7, and gal-9 [21, 25–27]. In fact, even mice that are genetically-deficient for both gal-1 and gal-3 appear normal [26]. Whether this is due the compensatory role of other galectins is a distinct possibility since multiple galectins are present in a given tissue, such as gal-1, gal-3, gal-8 and gal-9, in the case of the mammary gland [15, 18, 28].

### Gal-7 in mammary tumorigenesis

**Fig 3:**
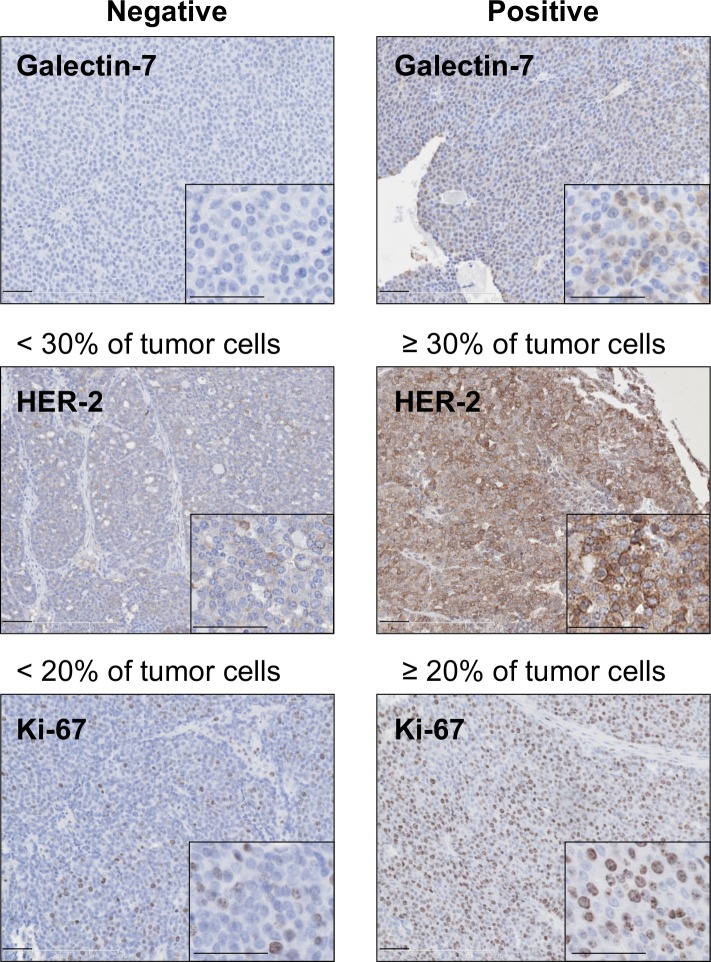
Immunohistological analysis of mammary tumour markers in presence or absence of gal-7. Immunohistochemical staining showing membrane-bound HER-2-positive cells and nuclear Ki67 expression in mammary tumors that were negative (*left panels*) or positive (*right panels*). Scale bars, 50 μm and 25 μm (insets).

**Fig 4:**
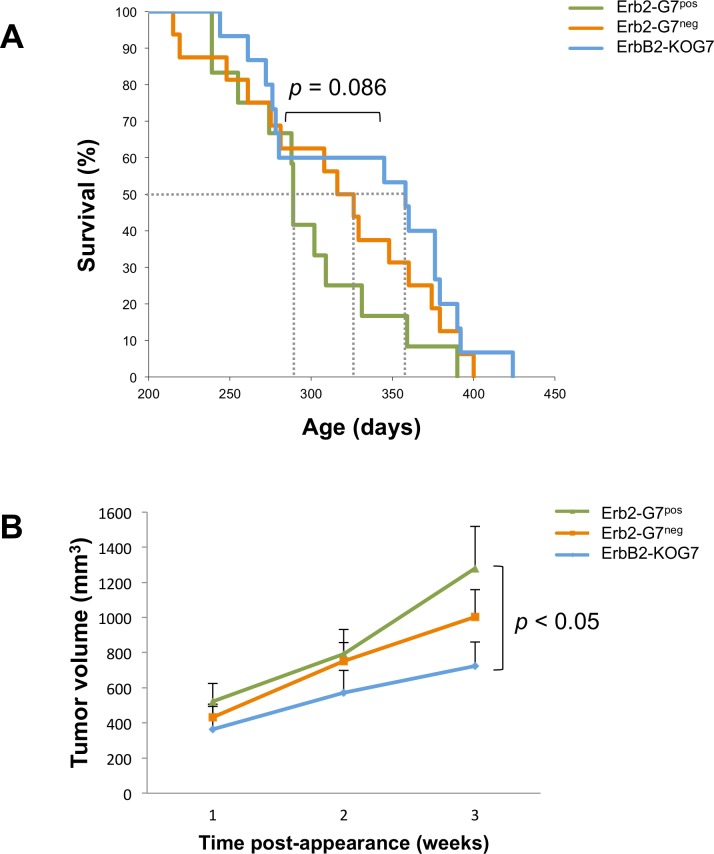
Decreased survival and higher tumor volume in presence of gal-7 in MMTV-ErbB2 mammary tumorigenesis. A) Kaplan-Meier survival curves for each group: G7+ (presence of gal-7 in mammary carcinoma; n = 12), G7- (absence of gal-7 in mammary carcinoma; n = 16) and KOG7 (lack of gal-7 throughout the body; n = 15). The median survival of each group is: 289 days (G7+), 316 days (G7-) and 358 days (KOG7). The *p* value 0.086 was calculated between G7+ and KOG7 groups. Comparison of G7- group to G7+ group (*p* = 0.231) or KOG7 group (*p* = 0.405). B) Average tumor volume of all mice from the specified group. The onset of the tumor was measured and identified as the first week post-appearance. The *p* value < 0.05 was calculated between G7+ (n = 11) and KOG7 (n = 13) groups, three weeks after tumor appearance. Comparison of G7- (n = 14) group to G7+ group (*p* = 0.33) or KOG7 group (*p* = 0.19).

Previous studies using gal-3-deficient mice have revealed a role for this galectin in breast cancer progression [29]. Thus, even though absence of gal-7 had no significant effect on the integrity of the mammary gland, we next examined whether it has a role in mammary tumorigenesis. For this purpose, we crossed MMTV-ErbB2 transgenic mice, a well-established preclinical model for HER-2 positive subtype of breast carcinoma [20], with gal-7-deficient mice for two generations in order to obtain mice that were gal-7-deficient (ErbB2-KOG7). Controls included mice that had at least one allele of wild-type gal-7 that were able to generate either gal-7-positive (ErbB2-G7^neg^) or gal-7-negative (ErbB2-G7^pos^) mammary tumors (**S1 Fig**). All mammary tumors were then collected and used to construct tissue microarrays (TMAs) in order to compare the expression of gal-7, HER-2, ER, and Ki-67 in each tumor by immunohistochemistry staining (**Fig 3**). Our analysis showed that As expected for MMTV-ErbB2 transgenic mice, ER status was negative for all tumors (*data not shown*) [30]. Our results showed that galectin-7-deficient mice generated a significant (*p* = 0.0076) lower frequency of mammary tumors expressing high levels of Her-2 (**Table 1**). This association between a low frequency of Her-2-positive tumors and low gal-7 expression was also observed when we compared gal-7-positive tumors and gale-7-negative tumors in mammary tumors generated in mice that contained at least one gal-7 wild-type allele (*p* = 0.0037). Kaplan-Meier survival curves also showed that mice with gal-7-positive mammary tumors had a significant (*p* = 0.086) lower median survival time (289 days) as compared to gal-7-negative tumors derived from ErbB2-KOG7 mice (358 days) (**Fig 4A**). Tumors that were gal-7-positive were also significantly (*p* < 0.05) larger than tumors of KOG7 mice (**Fig 4B**).

**Table 1:** Comparison between ErbB2 tumors induced in the presence or absence of gal-7 in mice.

Characteristics	Erb2-G7^pos^	ErbB2-KOG7	*p*	Erb2-G7^neg^	*p*
HER-2 membrane expression					
≤ 30% of tumor cells	1/10	5/6	0.0076	6/7	0.0037
> 30% of tumor cells	9/10	1/6		1/7	
Ki-67 expression					
< 20% of tumor cells	1/12	2/15	NS	7/15	0.0433
≥ 20% of tumor cells	11/12	13/15		8/15	

## Conclusions

In the present work, we have shown that: 1) the development of mammary epithelium appears normal in galectin-7-deficient mice; 2) absence of galectin-7 delayed the development of MMTV-ErbB2 mammary tumors, and 3) expression of gal-7 correlates with a higher frequency of mammary tumors with Her-2-positive tumors. Taken together, these results support the view that gal-7 plays an important role in primary breast cancer by accelerating tumor progression. They also confirm previous studies that linked gal-7 with the HER-2-positive molecular subtype. Whether expression of gal-7 and HER-2 are linked at the molecular level is an interesting possibility supported by preliminary findings using MCF-7 with or without HER-2 breast cancer cell lines. There again, we found that the presence of HER-2 in MCF-7 cells correlated with increased *gal-7* mRNA expression (**S2 Fig**). Such cell model will thus be useful to clarify a potential functional link between gal-7 and HER-2. Future studies will be necessary to establish the mechanism by which gal-7 accelerates the development of primary breast cancer in mice.

## Supporting Information

S1 Fig Experimental design. MMTV-ErbB2 transgenic mice were backcrossed with gal-7-deficient (KOG7) mice to generate gal-7-deficient MMTV-ErbB2 (ErbB2-KOG7) transgenic mice. Controls included mice that had at least one allele of wild-type gal-7 that were able to generate either gal-7-positive (ErbB2-G7^neg^) or gal-7-negative (ErbB2-G7^pos^) mammary tumors. PCR genotyping and immunohistochemistry were both used to distinguish between experimental and controls groups. Tumors were detected in mice with the *ErbB2* transgene only (Total period of observation: 425 days).
(TIFF)

S2 Fig mRNA upregulation of gal-7 by HER-2 in MCF-7 cells. A) mRNA levels of *gal-7* and *GAPDH* (as a loading control) were assayed by semi-quantitative RT-PCR in MCF-7 neo and MCF-7 HER-2 cells. B) Immunohistochemical staining on smear cells to confirm HER2 gene expression.MCF-7 transfectants were a generous gift from Dr. Mien-Chie Hung (University of Texas, MD Anderson Cancer center). Scale bars, 100 μm.
(TIFF)
