# Supplementary figures and images for: Galectin-7 Expression Potentiates HER-2-Positive Phenotype in Breast Cancer
Source: PLoS One. 2016 Nov 30;11(11):e0166731. doi: 10.1371/journal.pone.0166731 (PMC5130216; doi:10.1371/journal.pone.0166731)

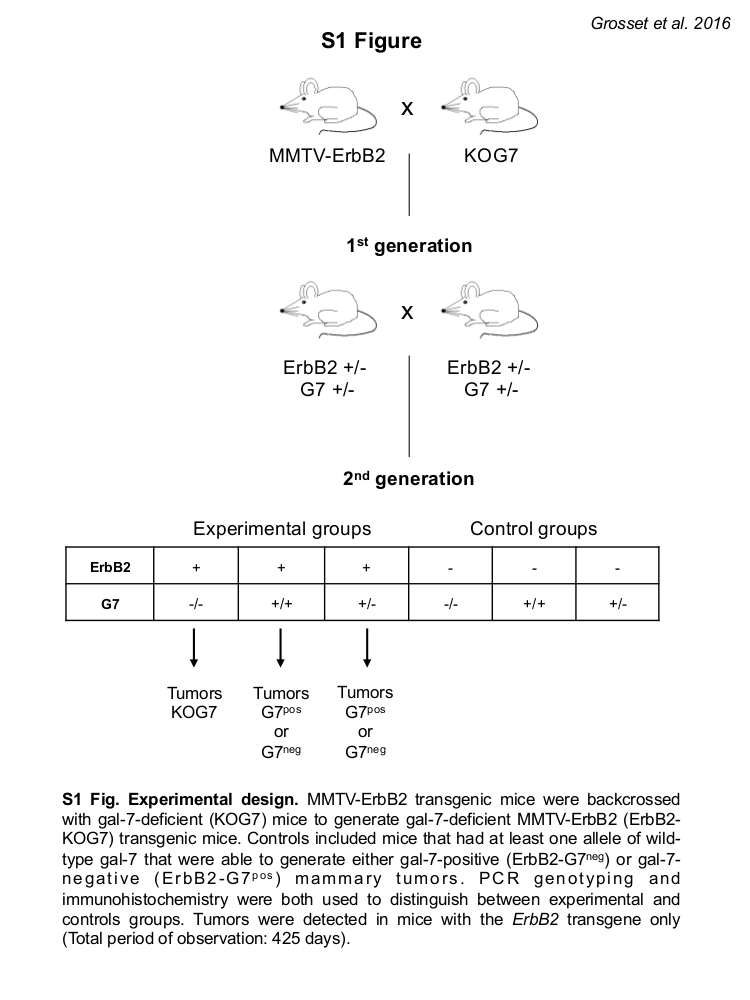

Supplement: S1 Fig — MMTV-ErbB2 transgenic mice were backcrossed with gal-7-deficient (KOG7) mice to generate gal-7-deficient MMTV-ErbB2 (ErbB2-KOG7) transgenic mice. Controls included mice that had at least one allele of wild-type gal-7 that were able to generate either gal-7-positive (ErbB2-G7neg) or gal-7-negative (ErbB2-G7pos) mammary tumors. PCR genotyping and immunohistochemistry were both used to distinguish between experimental and controls groups. Tumors were detected in mice with the ErbB2 transgene only (Total period of observation: 425 days). (TIFF) [file pone.0166731.s001.tiff]

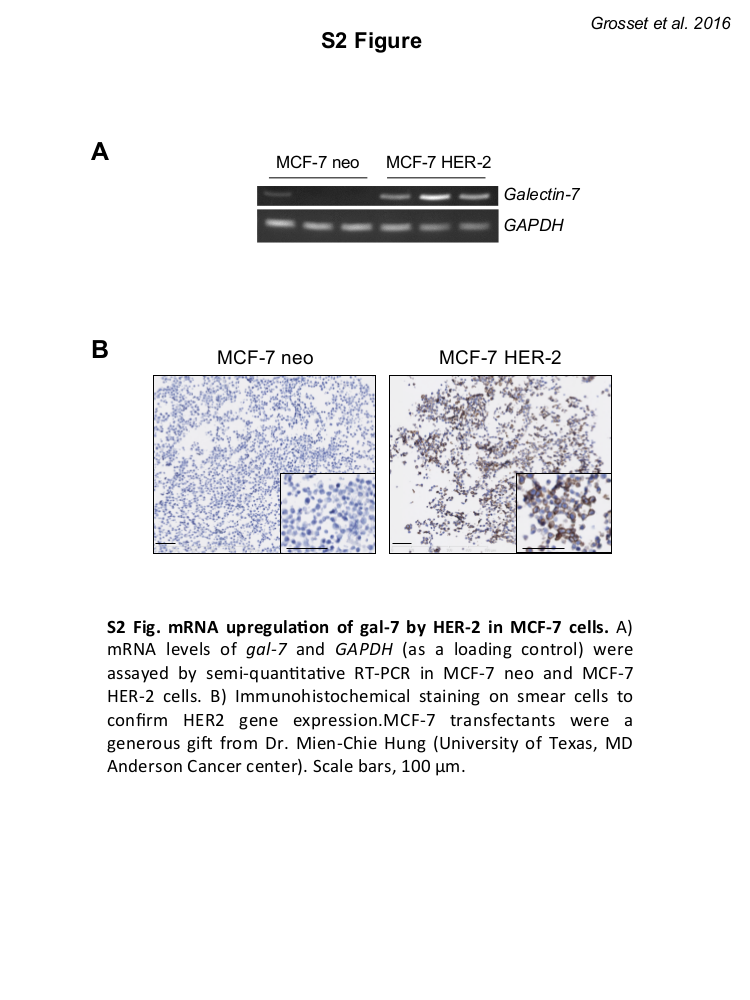

Supplement: S2 Fig — A) mRNA levels of gal-7 and GAPDH (as a loading control) were assayed by semi-quantitative RT-PCR in MCF-7 neo and MCF-7 HER-2 cells. B) Immunohistochemical staining on smear cells to confirm HER2 gene expression.MCF-7 transfectants were a generous gift from Dr. Mien-Chie Hung (University of Texas, MD Anderson Cancer center). Scale bars, 100 μm. (TIFF) [file pone.0166731.s002.tiff]
